# The Minamata Convention on Mercury: Time to Seek Solutions with Artisanal Mining Communities

**DOI:** 10.1289/ehp.1408514

**Published:** 2014-08-01

**Authors:** Samuel Spiegel, Susan Keane, Steve Metcalf, Marcello Veiga, Annalee Yassi

**Affiliations:** 1 Centre of African Studies, University of Edinburgh, Edinburgh, United Kingdom; 2 Natural Resources Defense Council, Washington, DC, USA; 3 Institute of Mining Engineering, and; 4 School of Population and Public Health, University of British Columbia, Vancouver, British Columbia, Canada

Gibb and O’Leary (2014) provided a timely review of the health effects of mercury in artisanal and small-scale gold mining (ASGM), calling for immediate implementation of the recently signed Minamata Convention on Mercury. They noted that Article 7 of the Convention [United Nations Environment Programme (UNEP) 2013] requires national action plans that include a public health strategy on mercury exposure in ASGM. However, although the analysis of Gibb and O’Leary reminds health officials about the hazards of mercury in ASGM, broader government policies toward poverty-driven ASGM must also change in order for these health hazards to be addressed.

One of the most critical shifts must be away from top-down mercury policy toward active engagement with ASGM communities to effectively address the underlying social and economic reasons why mercury is used. Notably, Annex C of the Convention (UNEP 2013) requires that governments devise strategies for involving stakeholders in developing national action plans. Currently, ASGM communities are widely excluded from health and environmental planning initiatives in many regions of Africa, South America, and Asia, inevitably leading to interventions that do not suit the communities’ realities (Clifford 2010; Hirons 2011; Spiegel et al. 2012). Public health professionals should encourage governments to pursue a participatory approach with ASGM communities to create successful local strategies for reducing mercury use.

Strategies should reflect lessons learned in past programs about the mining policies required for mercury reduction solutions to take hold in ASGM communities. The United Nations Industrial Development Organization (UNIDO) conducted mercury abatement programs in ASGM that yielded some positive results (Cordy et al. 2013), including programs in Sub-Saharan Africa supporting adoption of gold extraction technologies that significantly reduce mercury use employing low-cost alternative technologies (Chouinard and Veiga 2008; Spiegel and Veiga 2010). However, the widespread adoption of such technologies was often impeded by the lack of appropriate national policies toward ASGM. In many cases, artisanal miners responded well to training on low-mercury technologies, but they struggled to obtain official “legal” mining permits that would have facilitated making committed investments in these alternative practices. For example, Tanzania’s mining policies favor large companies, relegate small-scale mining to less desirable locations, and do not legally recognize most “artisanal mining” (Bryceson et al. 2014; UNEP 2012). In Mgusu (Tanzania), the UNIDO project sought to provide education to minimize mercury use in ASGM but was hindered by ambiguity over who had the “right to mine”—artisanal miners or a medium-scale mining company—preventing the long-term delivery of such initiatives (Spiegel 2009a).

To convey messages about mercury risks and mercury-free alternatives, community-based approaches can be more effective than conventional technical strategies that have dominated mercury-reduction initiatives. In Zimbabwe, UNIDO mercury abatement campaigns had some promising results in promoting cleaner technologies using such alternative approaches (Metcalf and Spiegel 2007). In Kadoma District, artisanal miners’ associations sought to raise awareness of mining communities’ rights while promoting education on mercury risk management strategies (Metcalf and Viega 2012). Working with a community theater group, a play was held to facilitate dialogue between artisanal miners, farmers, and others affected by mercury, adapting the narrative of Romeo and Juliet to illustrate tensions in the community about toxic risks from mining (Metcalf and Viega 2012).

Finally, public health officials and others should ensure that ambitious mercury reduction targets are not used as a rationale for harshly policing impoverished mining communities. The government of Zimbabwe implemented heavy-handed police crackdowns on ASGM between 2006 and 2009, which had negative environmental and social repercussions, weakening trust between regulators and low-income mining communities (Metcalf and Veiga 2012; Spiegel 2009b). More than 30,000 miners were arrested, some artisanal miners turned to working at night to avoid police, and some artisanal primary ore (land-based) miners turned to environmentally hazardous riverbed gold panning to evade surveillance, all resulting in worse public health and environmental outcomes.

The challenges of reducing mercury use in ASGM have long been documented, as noted previously (Kessler 2013; Schmidt 2012). We strongly agree with Gibb and O’Leary (2014) that national health campaigns—as required by the Minamata Convention—should be implemented immediately, but we advise governments to work on rectifying the inequities in mining policy needed to facilitate the shift away from mercury use. The World Federation of Public Health Associations has called on all governments and stakeholders to promote essential values of public health when implementing public policies—including solidarity, participation, empowerment, fairness, and social justice (Borisch 2012). Now that Gibb and O’Leary (2014) have synthesized the vast evidence confirming that mercury use in ASGM results in health impacts, countries should move toward working with ASGM communities to implement local solutions. Thus, we urge governments to focus on equity-sensitive approaches consistent with these essential values of public health.
